# Epidemiology of Injury-Related Death in Children under 5 Years of Age in Hunan Province, China, 2009–2014

**DOI:** 10.1371/journal.pone.0168524

**Published:** 2017-01-11

**Authors:** Xiong Lili, He Jian, Li Liping, Liu Zhiyu, Wang Hua

**Affiliations:** 1 Maternal and Children Health Care Hospital of Hunan Province, Changsha, China; 2 Injury Prevention Research Center, Shantou University Medical College, Guangdong Province, China; Centre Hospitalier Universitaire Vaudois, FRANCE

## Abstract

**Background:**

Injury is an important cause of childhood mortality in China. We described the epidemiology and trends of injury-related deaths of children <5 years of age in Hunan province, and discussed several policy implications.

**Methods:**

Injury-related deaths of children <5 years of age in 2009–2014 were identified from surveillance data. All specific injury mortality and mortality rates in urban and rural area were calculated from census data; Cochran-armitage trend test was used to assess the time trends.

**Results:**

Injury was the leading cause of death in children <5 years of age. Overall injury mortality was 48.96 per 100,000 persons, gradually declined with the year (*Z* = -18.75, *P*<0.001), and accounted for 27.14% of all deaths. Injury mortality in rural areas was 64.66 per 100,000 persons, which was more than 3.73 times higher than the rate in urban areas. The three leading causes of injury-related death were drowning (43.63%), suffocation (27.57%), and traffic accidents (14.34%). Suffocation was the leading cause in children <1 year of age (79.49%). Suffocation has high incidence in the winter and spring, and drowning has high incidence in the summer season. Drowning was the leading cause in children 1–4 years of age (62.80%). Drowning and suffocation accounted for 67.74% and 65.11%, of injury-related deaths that occurred at home; while the traffic injury deaths (54.12%) occurred mainly in transit.

**Conclusions:**

Injury-related fatalities in children <5 years of age followed time trends that were different in rural and urban areas. Effective childhood injury prevention may require different prevention policies combination depending on epidemiological characteristics such as development of injury surveillance and public education on injury knowledge. There is a need for evidence-based surveillance of risk factors for development of effective injury prevention programs.

## Introduction

Childhood injury is a global health problem, especially for low- and middle-income countries [1, 2]. The rate of childhood injury-related deaths is 3.4 times higher in low- and middle-income countries than in high-income countries [3, 4]. In 2013, accidental injuries were estimated to cause 324,000 deaths in children 1–59 months of age worldwide in 2013 [5]. China ranked the fifth in the number of deaths in children <5 years of age in 2013 [5], and injuries are now the fourth leading cause of death in China [1]. It is estimated that 11,000 infants and 20,000 children of 1–4 years age die each year in China based on the analysis of 2010 data. In 2014, injury-related deaths accounted for nearly 50% of all deaths in children <5 years of age in China [6]. The China National Program for Child Development (2010–2020) demands that injury-related mortality be reduced by one sixth of the 2010 level [7]. We should make every effort to achieve this requirement to control and reduce childhood injury-related mortality.

Sweden considers injury as a public health problem that society as a whole must control, and implemented a societal approach to the promotion of safety beginning in the 1950s. The program includes development of injury surveillance, public information and education, environmental improvements, and product safety development. It is widely believed that injury surveillance is a crucial prerequisite for effective injury prevention and control [8]. The Chinese Childhood Development Plan also specifies establishing child injury surveillance and an injury reporting system [7]. We should comply with the requirement to set up and implement effective surveillance and reporting.

Effective programs to prevent and control injury require accurate information on the extent and nature of common injuries and injury-related deaths. The usual sources of this information are vital statistics (e.g. death certificates or mortuary records), police reports of vehicle crashes and other events resulting in injury, and healthcare records. All of these sources have shortcomings related to lack of specific information, with the injury described only as the cause of death. Recent efforts have been made in China to improve the collection of injury data from several primary sources, such as community health clinics and hospitals, especially in Beijing, Shenzhen, Shanghai, and the provinces of Zhejiang and Fujian [9]. The National Maternal and Child Health Surveillance Point (HSP) system in Hunan province is the only source of surveillance data on mortality of children <5 years of age. The information referring to injury on the death case card of children under 5 years is only the name of the six injury-specific causes of death that are far less to comply with the required childhood development goals.

The goal of this study was to determine the epidemiology and trends of injury-related deaths in children <5 years of age from surveillance data obtained from 2009 to 2014 in Hunan province. We hope that the study will prompt timely investigation of injuries and surveillance including the distribution and causes of injury-related deaths as well as the key social influences. The surveillance system will inform the public education contents and support local laws and regulations intended to reduce injury-related childhood deaths. The epidemiological results of our study in Hunan province will also assist in the development of national childhood injury prevention strategies in China.

## Data and Methods

### Data sources

Mortality surveillance data were obtained from the National Maternal and Child HSP system, which includes children <5 years of age living in all 123 counties of Hunan province. The case surveillance data included information on the primary cause, date, and location of death, sex, and age. Causes of death were classified according to the World Health Organization International Classification of Diseases, Tenth Revision (ICD-10; World Health Organization, 1992). The six leading causes of injury were traffic injury (V01–V89, V99, and Y850), poisoning (X20–29, X40–49), drowning (W65–W74), suffocation (W79–80 and X47), and falls (W00–W19). Other causes and poorly-defined causes were recorded as “other”.

The population figures for children <5 years of age residing in urban and rural areas between 2009 and 2014 were obtained from the HSP Hunan province annual report. They were used to calculate cause-specific injury mortality rates and areas of residence injury mortality rates per 100,000 children between 2009 and 2014.

Hunan province is located in southeastern China, covers 21.18 km^2^, and has a population of 71.47 million people, 123 counties, and 14 cities. It has been in the HSP system since 1990, which now covers all 123 counties.

### Data quality control

Community health service centers in urban areas and health centers in rural areas are responsible for recording and verifying deaths of all children <5 years of age and reporting them to the district or county maternal and child health care institution, which checks the death case cards accuracy by checking with the rescue records in the hospital before death and completeness by checking the basic information as name, gender, age with the local disease prevention and control center monthly. The district or county maternal and child health care institution should also enter these death cards in the computer system and report to the municipal maternal and child health care institution and mail the paper case cards of all children <5 years of age with their hospital death certificates. Then, the municipal maternal and child health care institution report these data to the provincial maternal and child health care institution with the same process.

The district or county maternal and child health care institution with the health administrative department conduct a monthly audit of the death case cards by extracting 20% samples of the urban and rural centers to check whether there is any omission for the completeness and whether there is error for the accuracy of the data. The municipal and provincial maternal and child health care institution organize audit quality control quarterly and semi-annual respectively. The provincial maternal and child health care institution every month organize clinical experts to review every child death case especially the death cause diagnosis. The same routine is to the district or county maternal and child health care institution and municipal maternal and child health care institution. It guarantees the validity of the death case information.

### Statistical analysis

Data were exported to Microsoft Excel 2010 and analyzed with SAS Version 9.4. Rates and proportions were the main study indexes. Six injury- and region-specific mortality rates per 100,000 persons were also calculated. Chi-square tests were used to compare differences in injury-related death proportions in hospital, in transit, and at home. Time trends were calculated using Cochran-armitage trend test. *P*<0.05 was chosen as the level of statistical significance.

## Results

### Basic information of injury-related deaths in children <5 years of age

Between 2009 and 2014, a total of 11413 injury-related deaths were reported, accounting for 27.14% of all reported deaths in this population (Table 1). Overall, injury was the leading cause of death in children <5 years of age. Prematurity/ low birth weight was the second cause of death accounting for 12.6% (*N* = 5289). The third and the fourth cause of death was the pneumonia and congenital anomalies accounting for 11.7% and 11.7% (*N* = 4913 and *N* = 4900) respectively.

**Table 1:** Basic information of injury deaths under 5 years of age in Hunan province, China, 2009–2014.

Basic information		2009	2010	2011	2012	2013	2014
**Total children**		3537926	3690681	3913231	3982138	41064481	4081734
**Total number of death**		8562	8393	7542	7290	6153	4118
**Death caused by injury**		2014	2130	2079	2141	1829	1220
**Area**	**Rural**	1776	1886	1832	1892	1617	1070
	**Urban**	238	244	247	249	212	150
**Gender**	**Male**	1265	1275	1200	1290	1133	745
	**Female**	748	850	860	848	696	474
**Causes**	**Drowning**	858	942	868	908	846	557
	**Suffocation**	553	584	586	642	466	316
	**Traffic**	271	298	307	294	276	191
	**Fall**	117	105	104	113	120	72
	**Poisoning**	48	46	59	45	31	32
	**Others**	167	155	155	139	90	52

The overall injury-related death rate in this age group was 48.96 per 100,000 children. Of these, 6908 injury-related deaths were in boys (accounting for 28.14% of all male <5 deaths) and 4476 in girls (25.82% of female <5 deaths). There were 29 cases with sexual ambiguity. The proportions of injury-related deaths that were reported in boys and girls were significantly different (*χ*^*2*^ = 4.66, *P* = 0.03).

### Trends in injury-related and injury-specific mortality rates in rural and urban areas

**Fig 1:**
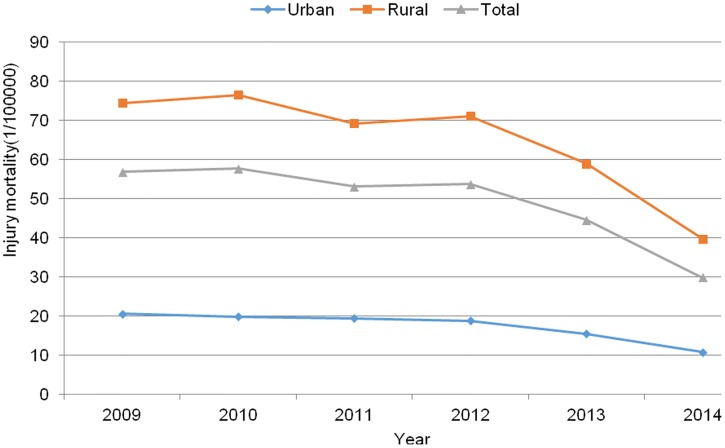
Trends of injury-related mortality in urban and rural areas and all of Hunan province, China, 2009–2014.

The overall injury-related annual mortality ranged from 29.89–57.71 deaths per 100,000 persons between 2009 and 2014. Cochran-armitage trend test showed a gradual decline in the total injury mortality, with the statistic *Z* value -18.75 and *P*<0.001. In rural areas, the overall rate was 64.66 per 100,000 persons, 28.30% of the rural total deaths, while the rural injury mortality was more than 3.73 times higher than that in urban areas of 17.33 per 100,000 persons, 20.75% of the urban total deaths (Fig 1). The proportions of injury-related deaths that were reported in urban and rural were significantly different (*χ*^*2*^ = 157.92, *P* <0.001).

**Fig 2:**
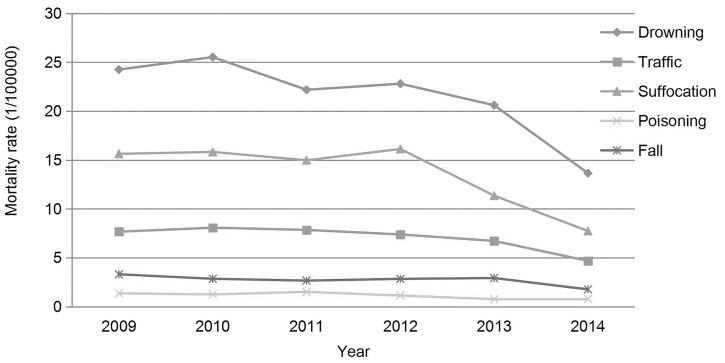
Trends of all-cause injury-specific injury mortality in Hunan province, 2009–2014.

The trends of cause-specific injury-related mortality rates are shown in Figure (Fig 2). Drowning had the highest rates (13.65–25.52 deaths per 100,000 persons) followed by suffocation (7.74–15.82 per 100,000 persons), traffic injury (4.68–8.07 per 100,000 persons), falls (1.76–3.31per 100,000 persons), and poisoning (0.75–1.51 per 100,000 persons) from 2009 to 2014.

### The leading injury-related causes of death

The leading cause of injury-related death was drowning, accounting for 41.75% to 46.25% of all injury deaths, followed by suffocation (25.48% to 29.99%), and traffic injury (13.46% to 15.66%) from 2009 to 2014 in Hunan province. The proportion of the top-three causes was jointly responsible for 83.52% to 87.21% of all injury deaths. Other causes, including falls, poisoning, and other accounted for 8.94% to 16.48% of all injury-related deaths. The proportions of the total number of deaths attributable to each of the leading causes of injury-related death changed significantly throughout the study period (*χ*^*2*^ = 64.12, *P* <0.001).

### Leading causes of injury-related death in different age groups

**Fig 3:**
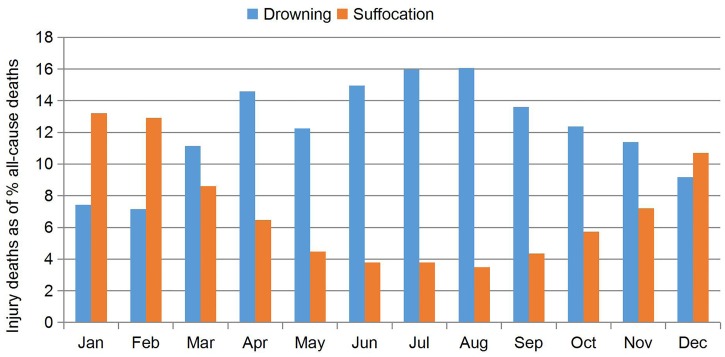
Monthly distribution of two injury-specific mortalities in Hunan province, 2009–2014.

The three leading causes of injury-related deaths in children <5 years of age were drowning, suffocation and traffic. Suffocation was the leading cause in neonates (88.31%) and infants (75.39%). The three leading causes in children 1–4 years of age were drowning (62.80%), traffic injury (16.73%), and suffocation (6.36%). A majority of deaths from all injuries (58.50%) occurred in children 1–4 years of age. Differences in the proportions of all the cause-specific injury-related deaths between age groups were significant (*χ*^*2*^ = 6259.7, *P*<0.001) (Table 2). Most suffocation (72.77%) and poisoning (63.98%) deaths were reported from October to March. Suffocation deaths was of 3.49% to 13.21% of all-cause deaths based on the month distribution, and drowning deaths was of 7.17% to 16.07% (Fig 3).

**Table 2:** Age group ranking of leading causes of injury-related death as proportions of all recorded deaths and proportions of all injury-related deaths in Hunan province, 2009–2014.

Age group	Injury death as of % total death	1	2	3	4	5	Others
**Neonate**	N = 1061	Suffocation	Drowning	Traffic	Poisoning	Fall	N = 70
	6.39%	N = 937,88.31%	N = 16,1.51%	N = 14,1.41%	N = 12,1.13%	N = 12,1.13%	6.60%
**Infant**	N = 2284	Suffocation	Traffic	Fall	Drowning	Poisoning	N = 176
	19.35%	N = 1722,75.39%	N = 128,5.60%	N = 104,4.55%	N = 89,3.90%	N = 65,2.85%	7.71%
**1-4y**	N = 6970	Drowning	Traffic	Suffocation	Fall	Poisoning	N = 425
	58.50%	N = 4377,62.80%	N = 1166,16.73%	N = 443,6.36%	N = 409,5.87%	N = 150,2.15%	6.10%
**0-5y**	N = 11413	Drowning	Suffocation	Traffic	Fall	Poisoning	N = 758
	27.14%	N = 4979,43.63%	N = 3147,27.57%	N = 1637,14.34%	N = 631,5.53%	N = 261,2.29%	6.64%

### Location of all-cause injury-related deaths

**Fig 4:**
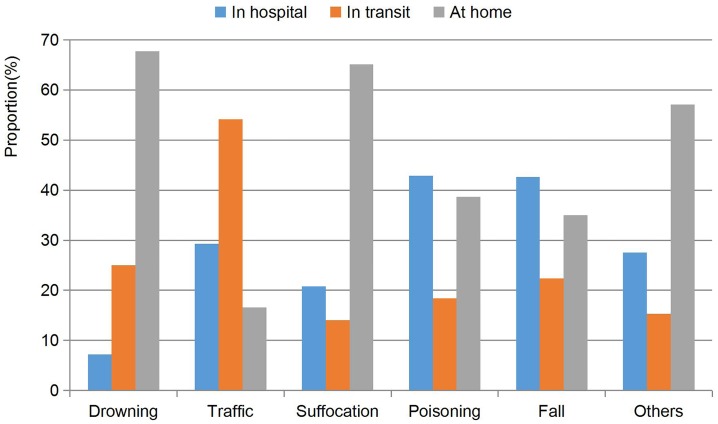
Location of injury-related deaths in Hunan province, China, 2009–2014 as a proportion of all injury-related deaths.

Most injury deaths occurred at home (56.51%), 25.21% occurred in transit, and 18.28% occurred in hospital. Most deaths from drowning (67.74%) and suffocation (65.11%) occurred at home, 54.12% of traffic injury deaths occurred in transit to the hospital; 42.91% of poisoning deaths and 42.63% of death from falls occurred in hospital. Most other injuries (57.12%) occurred at home (Fig 4). The proportions of injury-related deaths occurring at different locations were significantly different (*χ*^*2*^ = 2196.8, *P*< 0.001).

## Discussion

We studied the epidemiology and trends of injury of children <5 years of age in Hunan province. The results are of value to health officials to understand the scope of the injury problem and to begin working to establish an injury surveillance system for prevention and control of childhood injuries. This is the first provincial estimate of injury rates, and found that the injury-related mortality was 48.96 per 100,000, which is seven times that of Beijing city and three times than that of Guangzhou city [10, 11]. The injury-related death rate was higher than that of Pakistan, where it is estimated to be 37 per 100,000 per year for children between 1 and 5 years of age [12]. Similarly, the rate in Hunan province was five times higher than the 8.5 per 100,000 reported in high-income countries [13]. In our study, the proportion of injury-related deaths to all reported deaths was 23.52% to 29.73% from 2009 to 2014 in children <5 years of age and the total injury-related deaths accounted for 27.14% of all deaths reported in that age group. Injury was the first leading cause of death among children under 5 years of age. Injury has gradually become the main cause of death in low- and middle-income countries. However, the proportion of childhood injury deaths in Hunan province was lower than the national average of 49.00% [6]. In Hunan province, the public health problem presented by injuries is more serious than that in eastern cities, and in some developing countries, let alone developed counties.

While the higher proportion of deaths of boys than girls is consistent with other studies [14, 15], the interpretation is limited by the lack of injury-specific data related to the sex of the child in the maternal and child health annual report. It is widely believed that the injury-related death rate is higher in boys than that in girls, and that is supported by existing results of national studies [16, 17]. We thus believe that in the external environment, increased attention should be paid to injury prevention in boys relative to girls.

In our study, injury-related mortality was 3.75 times higher in rura areas than in urban areas, which was in line with the previous reports [12, 18]. This might be associated with the education level of parents and the availability of large spaces for activity in rural areas. No differences in nonfatal injuries to children <5 years of age in rural and urban areas have been reported [16, 17]. The results underscore the need for increased attention to injury prevention in rural, compared with urban areas.

We found a trend of decreasing injury mortality between 2009 and 2014. Our study results are consistent with reports of a significant, decreasing trend in overall and injury-related mortality in China and other countries [19–22]. However, childhood injury mortality has already declined by 50% in high-income countries under the effort of ensuring better nutrition and promoting safe motherhood for children aged 0 to 6 years old. The reducing incidence of injury in Hunan province was associated with the mandate that the original reporting data of deaths in children <5 years of age be imported into the HSP system. What is more, for the implementation of the national basic health service, the residents’ health records for children aged 0 to 6 years old are required to be established. From 2012, child care service qualified county had begun to be created and the project of nutrition improvement for children came into effect in Hunan province. From 2013, health care workers for children need to be trained in poor areas in Hunan province. These projects are helpful to promote the health conditions in children <5 years of age.

Suffocation was the leading cause of injury-related death in children <1 year of age (neonates and infants). It had a mortality of 11.40 per 100,000 persons and accounted for 79.49% of all injury deaths in the study popoulation, which is consistent with reports from other countries [19, 23, 24]. Suffocation due to lack of oxygen can have many causes. Accidental airway obstruction is a frequent cause of death in children <4 years of age [25]. It can be caused by plastic bags, pillows, oronasal obstruction by soft bedding, or respiratory tract obstruction by aspiration of foreign bodies [26]. Suffocation can also result from unsafe sleeping environments such as bed-sharing and sleeping with adults. Suffocation occurred mostly in the winter and spring. In cold weather, parents are fond of thick quilts and bed-sharing with infants. The American academy of pediatrics has identified bedding such as pillows, blankets, and quilts as potentially hazardous for the infant sleep environment. Bedding use is a modifiable risk factors for sudden infant death syndrome and unintentional sleep-related suffocation. Adults may lie on or roll over on top of or against an infant while sleeping. An adult may wedge and entrap an infant between two objects [22].

Our study found that 65.1% of children died of suffocation at home without receiving hospital care. In case of suffocation, most infants and the neonates die at home if parents do not know first-aid or live far away from a hospital. Health education about prevention and reducing exposure to conditions that increase the risk of suffocation should be promoted, in addition to education about parenting and simple emergency measures.

Drowning is the leading cause of death of children 1–4 years of age worldwide [22, 27], and the mortality of drowning was 18.68 per 100,000 persons in our study. Drowning accounted for 41.75% to 46.25% of all injury-related deaths overall, but in northern provinces such as Neimenggu, drowning is the third leading cause, responsible for 14.65% of all deaths between 2008 and 2014 [28]. In our study, drowning mortality was similar to that in Guangxi province, which borders the ocean, about 30 per 100,000 children 0–4 years of age [29]. In southern provinces, drowning mortality is probably related to exposure to open water. The drowning injury presented a high incidence in summer season. In most countries, drowning is among the three leading causes of death from accidental injury, and the rates are highest in children <5 years of age [12]. Because it is associated with childhood growth and development, most infants <1 year of age who drown were unattended, or attended by unqualified caregivers, such as younger brothers or sisters. [2]. Small children can drown in a few centimetres of water at the bottom of a bucket, in the bath, or in a rice field. We should strengthen the education of drowning risks and increase the safety awareness of parents, increase the safety precautions around dangerous water environments and swimming pools, and avoid exposing children to water where there is a drowning risk.

Mortality from traffic injury was 6.14 per 100,000 persons, and was the third leading cause of injury-related death. In the United States, the leading cause of death in children 1–4 years of age is traffic injury [30]. The risk of traffic injury increases with the age. Children under-5 years of age are difficult to be seen in traffic because of their short stature, and the primary injury is usually to the head. Children are prone to be injuried because their body and perception are not fully developed, and because they tend to ignore important risk factors. The parents’ perception of risk plays an important role in protecting children <5 years of age from the risks of traffic injury. We should strengthen traffic safety education of parents and require the use of proper child restraints. The use and correct installation of child restraint systems can reduce infant mortality by 70% and the mortality of children 1–4 years of age by 54% [2].

Most traffic injury deaths (54.12%) occurred in transit, which suggests that many injured children do not receive medical care [28]. The most critical problems related to prehospital and emergency care are the absence of a triage system and the long delay between the time of injury and reaching a hospital. The availability of good rehabilitation services is also an important requirement for the proper recovery of children following a road traffic injury. Most other injury deaths(57.12%) occurred at home, which explained that family injury prevention was important to reduce children <5 years of age from the risks of injury.

Our study has some limitations. Deaths of children <5 years of age are often under-reported by the The National Maternal and Child Health Surveillance Point (HSP) system, thus decreasing the accuracy of the data. The data were acknowledged as authoritative by various governmental organizations such as the Ministry of Health, the Provincial Bureau of Statistics and the Provincial Council Working Committee on Women and Children and released in China and abroad by the provincial government as indicators of the overall health of children in each province. The local government hopes that these data are favorable. However, with the provincial, municipal and county level data quality control and the review of the death cases, the overall data quality and credibility is feasible. Second, the cause of 6.64% of injury-related deaths could not be included with any of the five specific injuries, raileading to possible misclassification. Third, the study is subject to the limitation of recall and misclassification of the cause of death, especially for deaths due to violence or abuse.

Our findings have several policy implications. It is important to enhance the focus on childhood injury as a health issue and to integrate injury prevention efforts with a combination of education, environmental modification, and legislation, of which legislation and environmental modification are the most urgently needed for prevention children <5 years of age from injury in Hunan province. The first step is to establish a provincial injury surveillance system to identify and control emerging hazards to children. More attention should be given to injury prevention in rural areas and in boys. Injury is preventable, and several well-known interventions such as pool fencing and use of proper child restraints are readily available. Childhood deaths and injuries can be avoided through education programs and regulations. For the population density and management convenience in the community, the propaganda of injury knowledge in the community would be more conducive to prevent injury. Healthcare providers play a critical role in educating parents about safety issues especially drowning, suffocation, and traffic injury. An important prerequisite is establishment of an emergency care system to rescue injuried children.

## Conclusions

Although injury-realted fatalities in children <5 years of age have shown a decreasing trend, childhood injuries remain a substantial public health problem in Hunan province. The injury-related death rate was higher in rural than in urban areas. The proportion of childhood deaths was higher in boys than in girls, and the most common causes of injury-related death in all age groups were drowning, suffocation, and traffic injury. The leading injury in children <1 year of age was suffocation, and drowning in children 1–4 years of age. Most deaths from suffocation or poisioning occurred in winter and spring. The home was the most common location of drowning and suffocation, most children who died of traffic injuries died enroute to the hospital. There is a need to establish a system for surveillance of risk factors to assist developing an effective, evidence-based injury prevention program.
